# ERK5 Activation Is Essential for Osteoclast Differentiation

**DOI:** 10.1371/journal.pone.0125054

**Published:** 2015-04-17

**Authors:** Shigeru Amano, Yu-Tzu Chang, Yasuhisa Fukui

**Affiliations:** 1 Division of Microbiology and Immunology, Department of Oral Biology and Tissue Engineering, Meikai University School of Dentistry, Keyakidai, Sakado City, Japan; 2 National Health Research Institutes, Zhunan town, Miaoli County, Taiwan, Republic of China; Faculté de médecine de Nantes, FRANCE

## Abstract

The MEK/ERK pathways are critical for controlling cell proliferation and differentiation. In this study, we show that the MEK5/ERK5 pathway participates in osteoclast differentiation. ERK5 was activated by M-CSF, which is one of the essential factors in osteoclast differentiation. Inhibition of MEK5 by BIX02189 or inhibition of ERK5 by XMD 8-92 blocked osteoclast differentiation. MEK5 knockdown inhibited osteoclast differentiation. RAW264.7D clone cells, which are monocytic cells, differentiate into osteoclasts after stimulation with sRANKL. ERK5 was activated without any stimulation in these cells. Inhibition of the MEK5/ERK5 pathway by the inhibitors also blocked the differentiation of RAW264.7D cells into osteoclasts. Moreover, expression of the transcription factor c-Fos, which is indispensable for osteoclast differentiation, was inhibited by treatment with MEK5 or ERK5 inhibitors. Therefore, activation of ERK5 is required for the induction of c-Fos. These events were confirmed in experiments using M-CSF-dependent bone marrow macrophages. Taken together, the present results show that activation of the MEK5/ERK5 pathway with M-CSF is required for osteoclast differentiation, which may induce differentiation through the induction of c-Fos.

## Introduction

Osteoclasts are TRAP (Tartrate-resistant acid phosphate)-positive multinuclear cells [TRAP (+) MNCs] derived from monocyte/macrophage lineage cells via preosteoclasts, and they play an important role in bone resorption [1]. Many osteoclast precursor cell lines differentiate into osteoclasts in response to stimulation by M-CSF and sRANKL [1,2]. It has been reported that activation of NFκB and p38 MAP kinase, elevation of calcium levels, and induction of c-Fos are essential for osteoclast differentiation [2,3]. The NFκB and ERK pathways are activated by sRANKL and M-CSF stimulation, respectively. It is known that the induction of c-Fos is also required for differentiation [2,3].

Both M-CSF and sRANKL are required for M-CSF-dependent bone marrow macrophages (M-BMMs) and a new osteoclast precursor cell line, 4B12, to differentiate into TRAP (+) MNCs [4]. In contrast, it has been shown that monocytic RAW264.7D clone cells differentiate into osteoclasts in response to sRANKL stimulation [5–7].

As a member of the ERK family, ERK5 has a unique carboxyl-terminal tail, which can activate gene transcription [8]. ERK5 possesses both a nuclear localization signal (NLS) and a nuclear export signal (NES), which allows it to shuttle between the cytoplasm and the nucleus. ERK5 is phosphorylated by MEK5 and travels to the nucleus to activate the transcription of a number of genes involved in cellular differentiation [8].

In the present study, we report that ERK5 is activated by M-CSF in 4B12 cells and that ERK5 activation is essential for the differentiation of 4B12 cells into osteoclasts. We also demonstrate that ERK5 phosphorylation is important for the differentiation of RAW264.7D clone cells and M-BMMs.

## Materials and Methods

### Cell culture and reagents

The osteoclast precursor cell line, 4B12 [4], was maintained in α-Eagle's Minimum Essential Medium (α-MEM) containing 10% fetal bovine serum (FBS) and 30% calvaria-derived stromal cell conditioned media (CSCM) [4]. RAW264.7D clone cells were maintained in α-MEM containing 10% FBS [6]. Bone marrow cells were obtained by flushing the femurs of 6-week-old DDY male mice. For the formation of M-BMMs, stromal cells free bone marrow cells were cultured in the presence of M-CSF (10 ng/ml) for 7 days. M-BMMs were suspended in α-MEM containing 10% FBS, and used for various experiments. The ERK5 pathway inhibitors BIX02189 (MEK5 inhibitor) and XMD8-92 (ERK5 inhibitor) were purchased from Selleck Chemicals (Houston, TX) and MedChemexpress (Princeton, NJ), respectively. Mouse M-CSF (mM-CSF) and sRANKL were obtained from R&D Systems (Pittsburgh, PA).

### TRAP (+) MNC formation and TRAP-solution assays

Cells were fixed with 10% formalin-ethanol after cultivation with the samples, and then they were stained to detect TRAP. TRAP (+) MNCs were counted using a light microscope. The enzyme activity in a ten-fold dilution of the culture medium was measured using the TRAP-solution assay as previously described [4]. These results are expressed as the mean ± standard deviation (SD) of two separate experiments in sixplicate cultures (n = 6) (*, p < 0.05).

### Western blot analysis

Total proteins were extracted using Cell Lysis Buffer purchased from Cell Signaling Technology (Beverly, MA). The extracted proteins were separated by 10% SDS-PAGE under reducing conditions and transferred to nitrocellulose membranes. The membranes were then probed with anti-phospho-ERK5 and anti-ERK5 antibodies that were purchased from Cell Signaling Technology, anti-c-Fos antibody from Santa Cruz Biotechnology Inc. (Santa Cruz, CA), and anti-β-Actin pAb-HRP-DirecT from MBL, Nagano. Primary antibodies were detected using horseradish peroxidase-conjugated secondary antibodies and visualized using LumiGLO Reagent and Peroxidet purchased from Cell Signaling Technology.

### Viability of the cells

The 4B12 cells and M-BMMs (1×10^6^/well) were cultured in a 96-well flat-type Nunc plastic plate in α-MEM containing 10% FCS with or without test samples for 24 hours. The Fluo Cell Double Staining Kit (Molecular Biotechnology, Göttingen, Germany) was used to measure the viability according to the manufacturer’s instructions. The observed fluorescence was converted to a cell number using standard curves generated for both viable and dead cells. The results are expressed as the mean ± standard deviation (SD) of three separate experiments in sixplicate cultures. RAW264.7D clone cells were stained with trypan blue, and stained and unstained cells were counted by microscopye. The results represent the means of three independent experiments.

### Short-interfering RNA (siRNA) transfection and quantitative real-time RT-PCR (qRT-PCR)

The 4B12 cells were transfected with GFP siRNA or MEK5 siRNA purchased from Invitrogen using Amaxa nucleofection (Lonza, Cologne, Germany) according to the manufacturer's protocol. After 24 and 72 h, total cellular RNA was extracted using the ReliaPrep. RNA Cell Miniprep System purchased from Promega Corporation (Madison, WI). Two micrograms of total RNA was transcribed into cDNA in a total volume of 20 μl using random primers and the High Capacity cDNA Reverse Transcription Kit manufactured by Applied Biosystems (Foster City, CA) according to the instructions. The cDNA was used as a template in the qRT-PCR assay. qRT-PCR was performed using the Roche LightCycler 480 real-time PCR system with the FastStart Universal Probe Master rox supplied by Roche Applied Science (Indianapolis, IN)**.** Primers and FAM-labeled probes used in the quantitative RT-PCR for each gene were as follows: MEK5: forward primer (5’- AAGCAGCCCAAGGAGAGAC), reverse primer (5’ TTGAACTGCACGATGAATGG), probe (mouse universal probe Library #17, Roche); c-Fos forward primer (5’ GGGACAGCCTTTCCTACTACC), reverse primer (5’ AGATCTGCGCAAAAGTCCTG), probe (mouse universal probe Library #67). The 18S RNA was used as an endogenous control for normalization. The relative amounts of cDNA were calculated based on the relative quantification method.

### Statistics

The significance of differences between groups was determined by use of Student’s *t*-test. Values of p<0.05 were considered to indicate statistical significance.

## Results

### Phosphorylation of ERK5 was upregulated by M-CSF but not by RANKL

**Fig 1:**
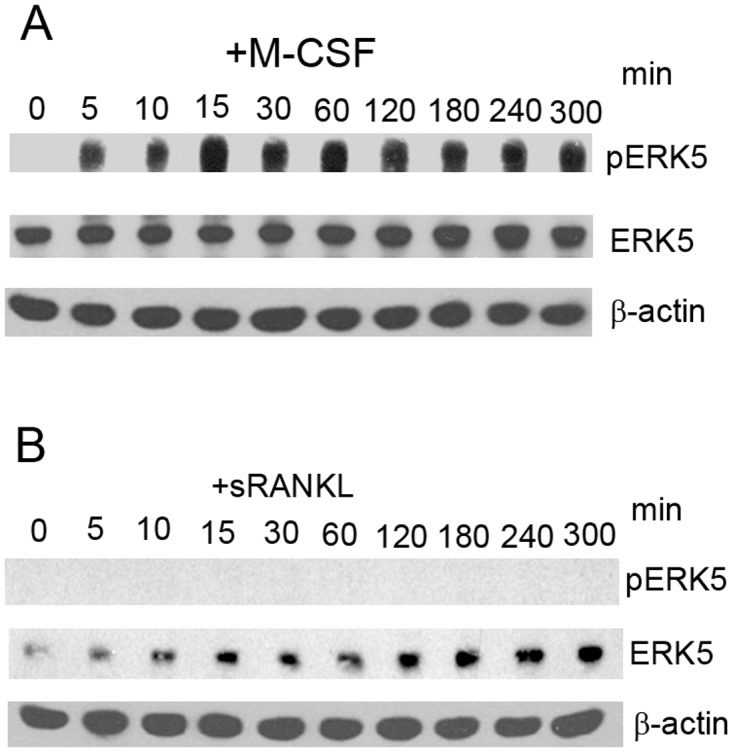
ERK5 was activated by M-CSF in 4B12 cells. (A, B) 4B12 cells were stimulated with 20 ng/ml M-CSF or 100 ng/ml sRANKL, and the phosphorylation of ERK5 was examined by Western blot analysis. Similar results were obtained in two independent experiments.

The activation of ERK5 in 4B12 cells treated with M-CSF or sRANKL was examined. ERK5 was activated by M-CSF but not by sRANKL. The ERK5 activation was detected as early as 5 min after M-CSF stimulation and continued for a long duration (Fig 1A and 1B).

### Inhibition of the MEK5/ERK5 pathway blocked osteoclast differentiation

**Fig 2:**
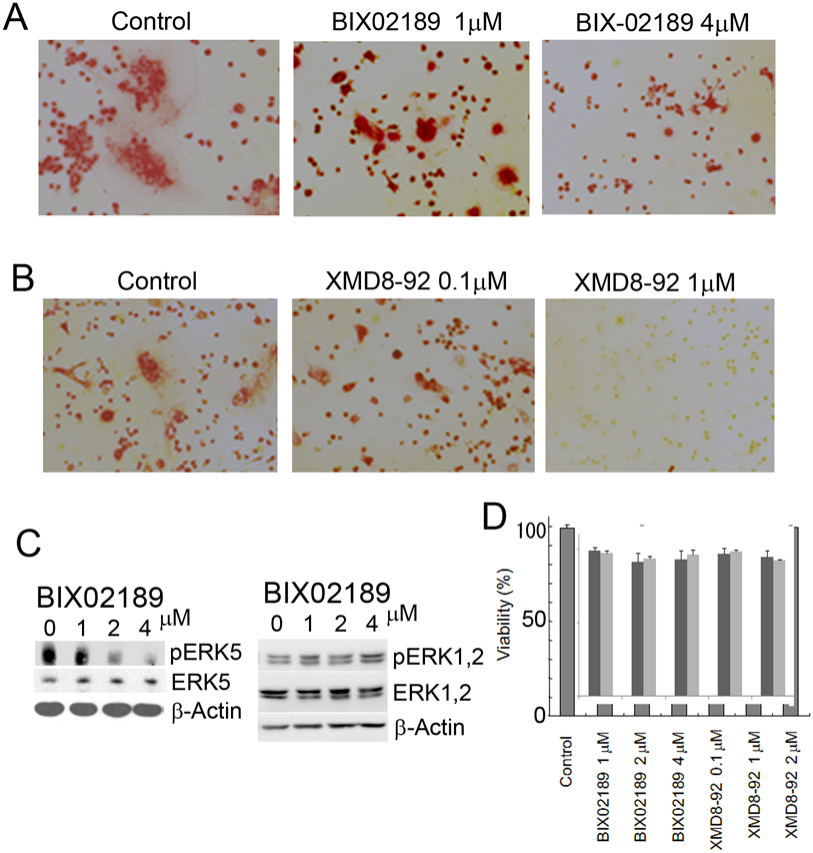
The formation of TRAP (+) MNCs in 4B12 cells was inhibited by BIX02189 and XMD8-92. (A) 4B12 cells were cultured with 10 ng/ml M-CSF and 10 ng/ml sRANKL in the presence or absence of BIX02189. After 7 days, the cells were fixed and stained to detect TRAP. (B) An experiment similar to A was conducted with XMD8-92. (C) The inhibition of ERK5 (left panel) or ERK1, 2 (right panel) phosphorylation by BIX02189 was examined by Western blot analysis. (D) The cell viabilities during the experiments were analyzed. Cells were incubated with the drugs for one day. Similar results were obtained in three independent experiments.

**Fig 3:**
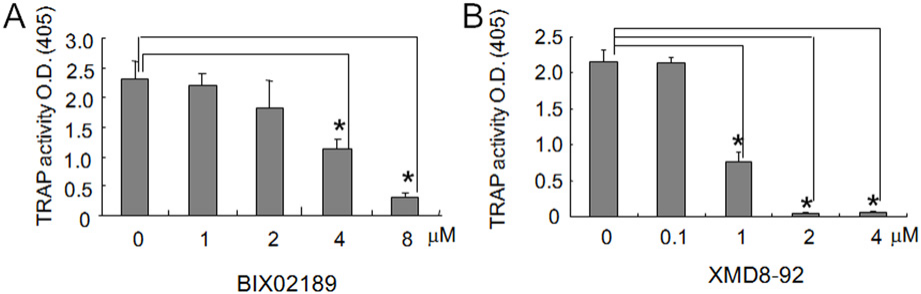
TRAP activity in 4B12 cells was inhibited by BIX02189 or XMD8-92. (A, B) 4B12 cells (5 × 10^3^) were treated with BIX02189 (A) or XMD8-92 (B) in the presence of M-CSF (10 ng/ml) and sRANKL (10 ng/ml). After 6 days, the TRAP activity in 4B12 cells was measured. Similar results were obtained in three independent experiments. *P<0.05 compared with the culture without inhibitors.

As mentioned above, 4B12 cells differentiate into osteoclasts in the presence of M-CSF and sRANKL. We tested the effects of the MEK5/ERK5 pathway inhibitors BIX02189 and XMD8-92 on osteoclast differentiation using 4B12 cells. BIX02189 and XMD8-92 inhibited the formation of TRAP (+) MNCs (Fig 2A and 2B). BIX02189 inhibited the phosphorylation of ERK5 but not of ERK1/2 (Fig 2C). The doses of BIX02189 and XMD8-92 had no effect on the cell viability of 4B12 cells (Fig 2D). As anticipated, TRAP activity in 4B12 cells stimulated with M-CSF and sRANKL was reduced in response to the treatment with BIX02189 or XMD8-92 (Fig 3A and 3B). The TRAP activity was partially inhibited by BIX02189 and was completely inhibited by XMD8-92.

**Fig 4:**
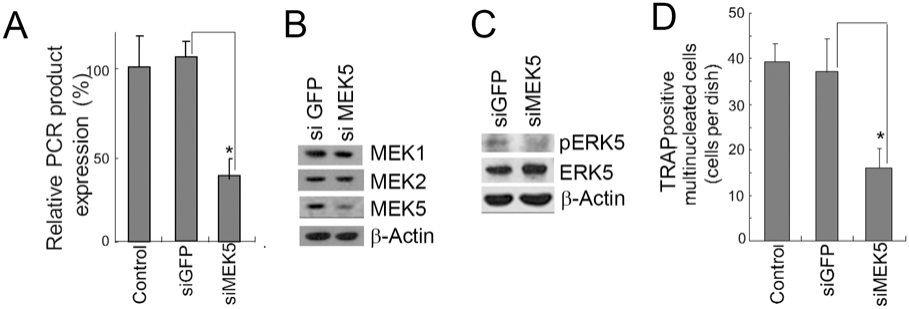
MEK5 siRNA inhibited the formation of TRAP (+) MNCs in 4B12 cells. (A) 4B12 cells were transfected with GFP siRNA or MEK5 siRNA. The 4B12 cells (2.5 × 10^4^) were cultured in the presence of M-CSF (10 ng/ml) and sRANKL (20 ng/ml). After 3 days, MEK5 gene expression was measured by qRT-PCR. Similar results were obtained in two independent experiments. *P<0.05 when compared with 4B12 cells transfected with GFP siRNA. (B) 4B12 cells (5 × 10^5^) were transfected with siRNAs for GFP or MEK5. The levels of MEK1, 2, and 5 were analyzed by Western blot analysis. Similar results were obtained in two independent experiments. (C) 4B12 cells were transfected with siRNAs for GFP or MEK5. The cells were stimulated with M-CSF (10 ng/ml). After 30 min, the phosphorylation of ERK5 was monitored. Similar results were obtained in two independent experiments. (D) 4B12 cells (5 × 10^5^) were transfected with GFP or MEK5 siRNAs. The transfected cells (5 × 10^3^) were cultured in the presence of M-CSF (10 ng/ml) and sRANKL (20 ng/ml). After 6 days, the number of TRAP-positive MNCs was counted. Similar results were obtained in three independent experiments. *P<0.05 when compared with 4B12 cells transfected with GFP siRNA.

We also tested the effects of MEK5 siRNA on the differentiation of 4B12 cells into TRAP (+) MNCs. Knockdown of MEK5 using a MEK5-specific siRNA selectively inhibited the expression of MEK5 at the mRNA and protein levels, but it did not affect the levels of MEK1 and MEK2 (Fig 4A and 4B). After M-CSF treatment, the phosphorylation of ERK5 was inhibited by MEK5 knockdown (Fig 4C). As expected, MEK5 knockdown suppressed the formation of TRAP (+) MNCs (Fig 4D). These results suggest that the MEK5/ERK5 pathway is required for the differentiation of 4B12 cells into osteoclasts.

### ERK5 activation was required for the induction of c-Fos

**Fig 5:**
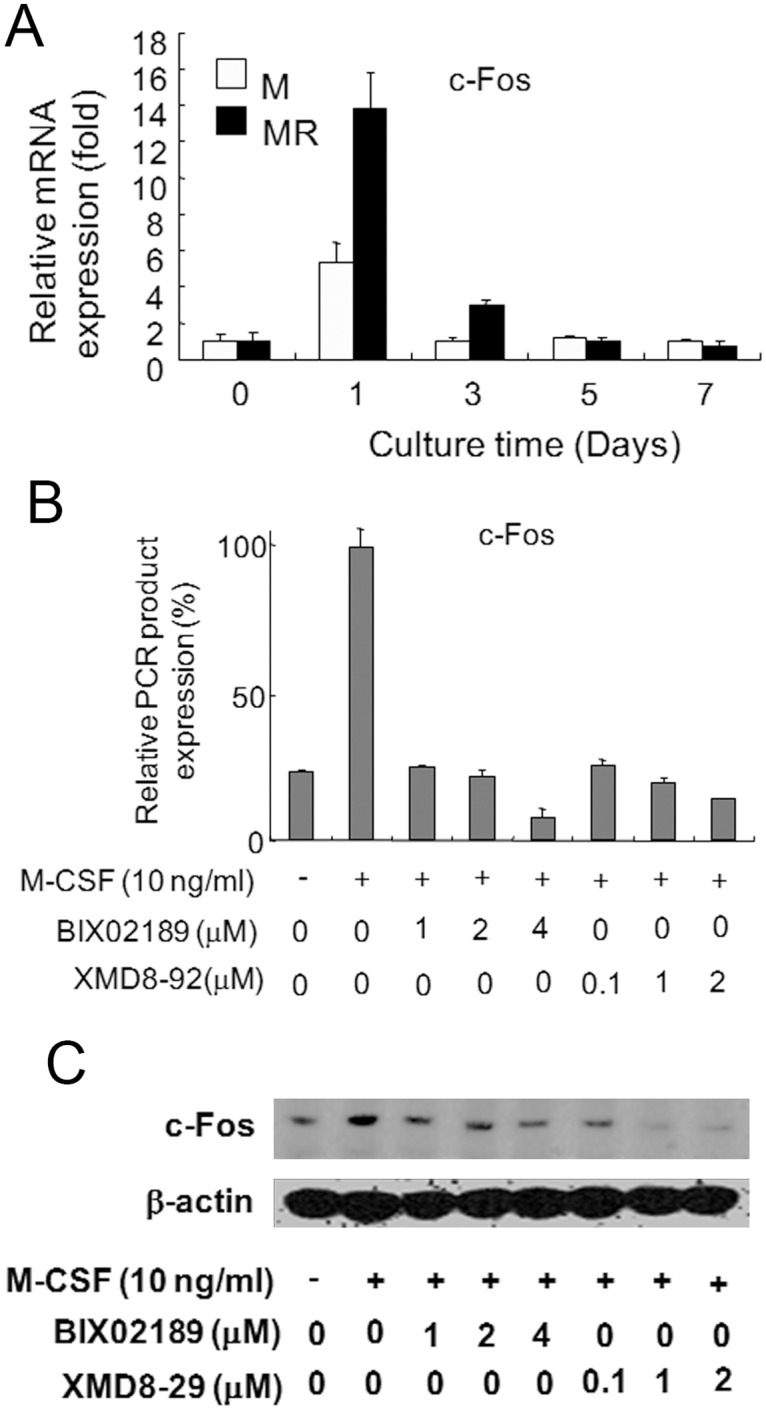
The induction of c-Fos was blocked by inhibition of the ERK5 pathway. (A) 4B12 cells (2.5 × 10^4^) were cultured with M-CSF (10 ng/ml) alone (M) or with M-CSF (10 ng/ml) and sRANKL (10 ng/ml) (MR). After the indicated times, c-Fos gene expression was measured by qRT-PCR. (B, C) 4B12 cells (2.5 × 10^4^) were pretreated with or without BIX02189 or XMD8-92 for 6 hrs and then cultured in the presence of M-CSF (10 ng/ml) for 24 hrs. The expression of c-Fos was measured by qRT-PCR and Western blot analysis. Similar results were obtained in two independent experiments.

Given that ERK5 can significantly induce c-Fos, we tested whether the induction of c-Fos in 4B12 cells was affected by ERK5 activation [8,9]. c-Fos mRNA expression was induced in 4B12 cells in response to stimulation with M-CSF alone, and this expression was enhanced following stimulation with M-CSF and sRANKL (Fig 5A). Pretreatment with both BIX02189 and XMD8-92 significantly inhibited M-CSF-induced c-Fos expression at the mRNA and protein levels (Fig 5B and 5C). These results suggest that ERK5 activation is required for the induction of c-Fos.

### The differentiation of RAW264.7D clone cells was blocked by inhibition of the ERK5 pathway

**Fig 6:**
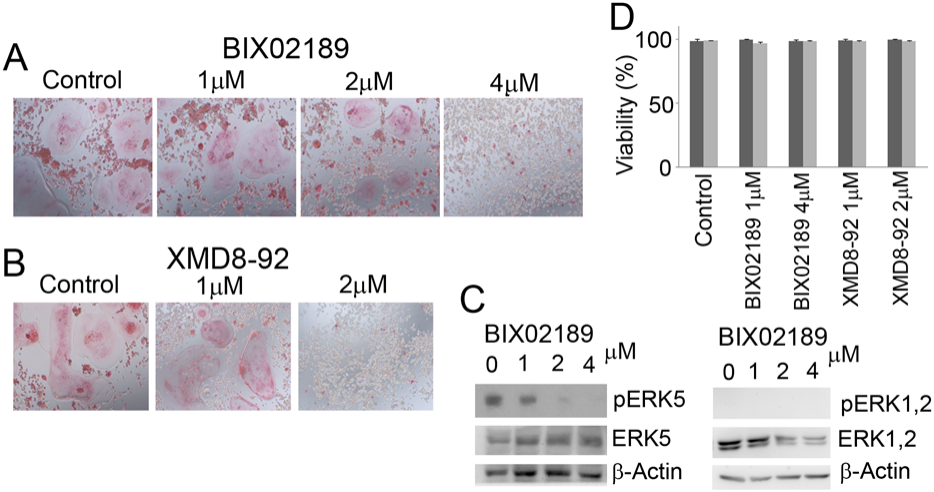
The formation of TRAP-positive MNCs in RAW264.7D clone cells was inhibited by BIX02189 or XMD8-92. (A) RAW264.7D clone cells were cultured with 50 ng/ml sRANKL in the presence or absence of BIX02189. The formation of TRAP-positive MNCs was inhibited when the concentration of BIX02189 reached 4 μM. (B) An experiment similar to A was conducted with XMD8-92. (C) The total proteins were extracted from the cells treated with BIX02189 for 6 hrs, and the phosphorylation of ERK5 and ERK1,2 was analyzed by Western blot analysis. (D) Cell viabilities during the experiments were analyzed. Cells were incubated with drugs for 1 day (dark gray bars) or 2 days (light gray ones).

**Fig 7:**
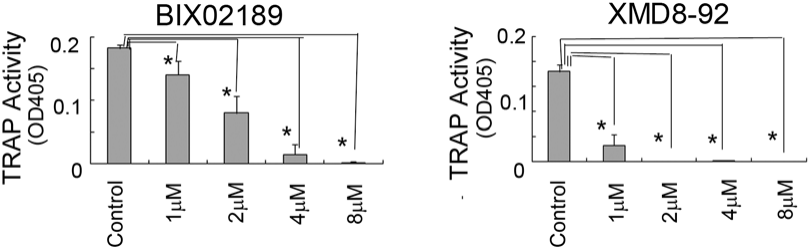
The TRAP activity in Raw264.7D clone cells was inhibited with BIX02189 or XMD8-92. RAW264.7D clone cells (5 × 10^3^) were treated with BIX02189 (A) or XMD8-92 (B) in the presence of sRANKL (10 ng/ml). After 6 days, the TRAP activity was measured. Both BIX02189 and XMD8-92 inhibited TRAP activity in RAW264.7D clone cells. *P<0.05 compared with the culture without inhibitors.

The differentiation of RAW264.7D clone cells was also activated by sRANKL, and M-CSF was not required for this process. The behavior of the RAW264.7D clone cells was examined during MEK5/ERK5 pathway inhibition. Inhibition of the MEK5 pathway reduced the formation of TRAP-positive MNCs (Fig 6A). Inhibition of the ERK5 pathway also reduced it (Fig 6B). ERK5 was constitutively activated (see the control lane in the Figure), and the activation of ERK5 was inhibited by BIX02189 (Fig 6C). ERK1 and 2 were not inhibited in these cells (Fig 6C). The viability of the cells was not affected by the drug treatments (Fig 6D). MEK5/ERK5 pathway inhibitors also reduced TRAP activity (Fig 7), which suggested that activation of the ERK5 pathway was required for the differentiation of RAW264.7D clone cells.

### The activation of ERK5 was required for the induction of c-Fos in RAW264.7D clone cells

**Fig 8:**
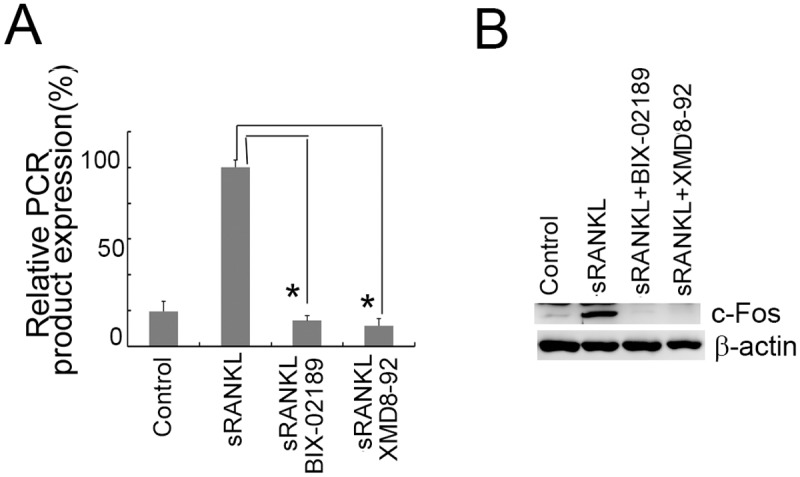
The induction of c-Fos in RAW264.7D clone cells was inhibited by BIX02189 or XMD8-92. (A) RAW264.7D clone cells (2.5 × 10^4^) were cultured in the presence of sRANKL (50 ng/ml) with or without BIX02189 or XMD8-92. After 20 hrs, c-Fos gene expression was measured by qRT-PCR. Similar results were obtained in two independent experiments. *P<0.05 when compared with the culture with sRANKL. (B) RAW264.7D clone cells (2.5 × 10^4^) were cultured with sRANKL (50 ng/ml). Subsequently, 5 μM BIX02189 or 1μM XMD8-92 was added to the samples, and c-Fos gene expression was examined after 6 hrs by Western blot analysis.

The induction of c-Fos was also examined in RAW264.7D clone cells. Similarly to the 4B12 cells, c-Fos was induced in RAW264.7D clone cells in response to sRANKL stimulation. This phenomenon was blocked by BIX02189 or XMD8-92 in RAW264.7D clone cells (Fig 8), suggesting that activation of the ERK5 pathway was required for the induction of c-Fos.

### The MEK5/ERK5 pathway was also involved in the differentiation of M-BMMs into osteoclasts

**Fig 9:**
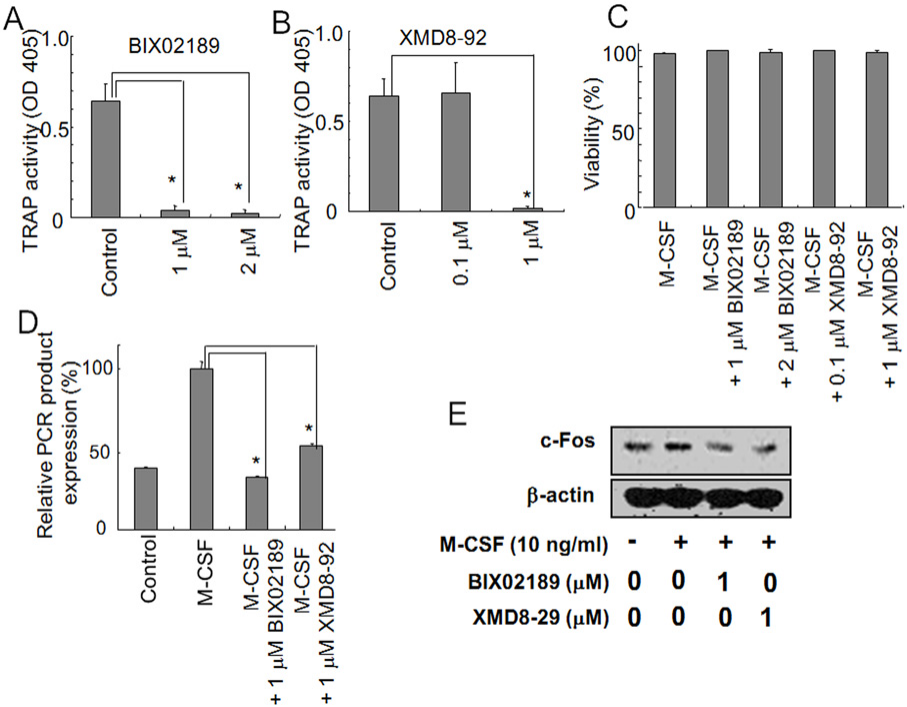
The formation of TRAP-positive MNCs in M-BMMs was also inhibited by BIX02189 or XMD8-92. (A, B) M-BMMs were pretreated with or without BIX02189 (A) or XMD8-92 (B) for 6 hrs and then cultured in the presence of M-CSF (10 ng/ml) and sRANKL (10 ng/ml) for 7 days. The TRAP activity in culture medium diluted ten times was measured. Results are expressed as the mean ± SD of sixplicate cultures. Significant differences were examined using the Student’s t-test, *P<0.05 compared with the culture without BIX02189 or XMD8-92. (C) The viability of the cells during the experiment was measured. Similar results were obtained in two independent experiments. (D, E) The induction of c-Fos was monitored at the mRNA and protein levels. Similar results were obtained in two independent experiments. Significant differences were examined using the Student’s t-test, *P<0.05 compared with the culture with M-CSF.

Finally, we examined the effect of inhibition of the MEK5/ERK5 pathway on the differentiation of M-BMMs into osteoclasts. M-BMMs were stimulated with M-CSF and sRANKL for 7 days to form TRAP (+) MNCs. MEK5 and ERK5 inhibitors blocked the formation of TRAP (+) MNCs, respectively (Fig 9A and 9B). These cells were more sensitive to the drugs than 4B12 or RAW264.6D clone cells. The viability of the cells was not affected by the drug treatment (Fig 9C). The induction of c-Fos was also examined. c-Fos expression induced by M-CSF was inhibited by treatment with the MEK5 and ERK5 pathway inhibitors (Fig 9D). These results suggest that M-CSF stimulation of the MEK5/ERK5 pathway is required for the formation of osteoclasts in primary M-BMM as well as in 4B12 or RAW264.7D clone cell cultures.

## Discussion

Numerous pathways are involved in the differentiation of osteoclasts; however, the role of the ERK pathway in osteoclast differentiation remains largely unknown. It has been shown that the inhibition of MEK1 and MEK2 promotes preosteoclast differentiation [10]. In the present study, we showed that the ERK5 pathway was important for osteoclast differentiation. We found that the MEK5/ERK5 pathway inhibitors BIX02189 and XMD8-92, as well as MEK5 siRNA, significantly inhibited osteoclast differentiation. However, BIX02189 partially inhibited osteoclast differentiation, and its inhibitory effects were weaker than those of XMD8-92. We propose that the MEK5/ERK5 pathway is a novel target for the suppression of osteoclast differentiation. In addition, we found that the induction of c-Fos was blocked by the inhibition of ERK5. A previous study has shown that mice lacking the proto-oncogene c-Fos develop bone disease osteopetrosis [11].

The activation of ERK5 may play an important role in the differentiation of preosteoclasts into osteoclasts by inducing c-Fos. In addition, cross talk may occur between the NF-κB and the ERK5 signaling pathways; however, this hypothesis requires further investigation.

ERK5 was constitutively activated in Raw264.7D clone cells. The activation of ERK5 was blocked when the RAW264.7D clone cells were cultured in serum-free medium (data not shown). It is likely that ERK5 in RAW264.7D clone cells is activated by some uncharacterized molecules that are present in the serum. We are currently searching for potential molecular stimulators of the ERK5 pathway in the serum.

Taken together, our results suggest that the ERK5 pathway is an important component in the signaling that leads to osteoclast differentiation.
